# *Plasmodium berghei *ANKA infection increases Foxp3, IL-10 and IL-2 in CXCL-10 deficient C57BL/6 mice

**DOI:** 10.1186/1475-2875-10-69

**Published:** 2011-03-28

**Authors:** Bismark Y Sarfo, Nana O Wilson, Vincent C Bond, Jonathan K Stiles

**Affiliations:** 1 Department of Microbiology, Biochemistry and Immunology, Morehouse School of Medicine, 720 Westview Drive, South West, Atlanta Georgia, GA 30310, USA

## Abstract

**Background:**

Cerebral malaria (CM) is a major cause of malaria mortality. Sequestration of infected red blood cells and leukocytes in brain vessels coupled with the production of pro-inflammatory factors contribute to CM. CXCL-10 a chemokine that is chemotactic to T cells has been linked to fatal CM. Mice deficient for CXCL-10 gene are resistant to murine CM, while antibody ablation of CXCL-10 enhanced the production of regulatory T cells (CD4+Cd25+Foxp3+) and IL-10 which regulate the immune system. Interleukin-2 (IL-2), a pro-inflammatory cytokine implicated in malaria pathogenesis has also been shown to be a key regulator of Foxp3. However the role of Foxp3 in resistant murine CM is not well understood.

**Methods:**

The hypothesis that resistance of CXCL-10-/- mice to murine CM may be due to enhanced expression of Foxp3 in concert with IL-10 and IL-2 was tested. CXCL-10-/- and WT C57BL/6 mice were infected with *Plasmodium berghei *ANKA and evaluated for CM symptoms. Brain, peripheral blood mononuclear cells (PBMCs) and plasma were harvested from infected and uninfected mice at days 2, 4 and 8. Regulatory T cells (CD4+CD25+) and non-T regs (CD4+CD25-) were isolated from PBMCs and cultured with *P. berghei *antigens in vitro with dendritic cells as antigen presenting cells. Regulatory T cell transcription and specific factor Foxp3, was evaluated in mouse brain and PBMCs by realtime-PCR and Western blots while IL-10, and IL-2 were evaluated in plasma and cultured supernatants by ELISA.

**Results:**

Wild type mice exhibited severe murine CM symptoms compared with CXCL-10-/- mice. Foxp3 mRNA and protein in brain and PBMC's of CXCL-10-/- mice was significantly up-regulated (p < 0.05) by day 4 post-infection (p.i) compared with WT. Plasma levels of IL-10 and IL-2 in infected CXCL-10-/- were higher than in WT mice (p < 0.05) at days 2 and 4 p.i. Ex-vivo CD4+CD25+ T cells from CXCL-10-/- re-stimulated with *P. berghei *antigens produced more IL-10 than WT CD4+CD25+ T cells.

**Conclusion:**

The results indicate that in the absence of CXCL-10, the resulting up-regulation of Foxp3, IL-10 and IL-2 may be involved in attenuating fatal murine CM.

## Background

Cerebral malaria (CM) is a major cause of malaria mortality in endemic countries. The current paradigm of CM pathogenesis suggests that parasite proliferation activates endothelial cells to produce adhesion molecules that enable sequestration of infected and uninfected red blood cells (RBCs) in brain capillaries which obstruct brain microvessels which results in severe inflammatory processes that lead to CM syndrome. Recently it has been reported that regulatory inflammatory responses are associated with CM but the mechanism by which they regulate CM pathogenesis is unclear. For example mice lacking T-cells do not develop CM, and CM is attenuated when CD4+ and CD8+ T-cells are blocked [1]. Thus, blocking activation of T cells appears to decrease the risk of CM severity.

The suppressive mechanism by which T- cells control CM pathogenesis is not fully appreciated. Regulatory T-cells (T-regs;CD4+CD25+Foxp3+) are a class of T-cells which limit excessive activation of T-cells, and overproduction of pro-inflammatory factors, and have been implicated in many infectious diseases but their role in CM immunopathogenesis is unclear. Recent reports indicate that chemokines involved in the recruitment of T-cells, during CM pathogenesis also recruit CD4+CD25+Foxp3+ [2-8]. For instance, increased level of the interferon-inducible protein-10 (CXCL-10) in CM patients is tightly associated with the fatal CM phenotype in children [5]. CXCL-10 and its receptor CXCR3 are required for the development of murine CM [6]. Also, CXCL-10 is specific for effector T-cell recruitment during infection and, therefore, its deletion reduces T-cell trafficking, which enhances production of CD4+CD25+Foxp3+ and suppressive cytokines interleukin-10 (IL-10) and tumor growth factor- β1 (TGF-β1) [9]. Interestingly, loss of CD4+CD25+Foxp3+ during simian immunodeficiency viral infection for example has been linked to changes in the expression of CXCL-10 [10].

Interleukin-2 (IL-2), which is produced by Th1 cells, is one of the potent inflammatory cytokines mediating multiple immune responses on activated B cells, monocytes, and natural killer (NK) cells. Treatments of *Plasmodium yoelii *17XL infected mice with this cytokine resulted in accumulation of T cells in the brain making resistant mice susceptible to CM [11]. Interleukin-2 is a key regulator of Foxp3, a specific marker for CD4+CD25+Foxp3+.

During *Plasmodium *infection, the balance between host pro-inflammatory and anti-inflammatory immune responses play important roles in pathogenesis of CM. A weak pro-inflammatory response may lead to persistence and replication of parasites while an excessive pro-inflammatory response may result in immunopathological consequences such as CM. Therefore, identifying regulatory mechanisms that control CM pathogenesis is important in developing interactions for CM. Regulatory T-cells (CD4+CD25+Foxp3+) are specialized T-cells which maintain immune system homeostasis. They constitute approximately 10% of host T-cells population [12,13]. Regulatory T-cells suppress activation and recruitment of T cells via production of suppressive cytokines such as IL-10 and TGF-β1 [14,15], to limit the ability of dendritic cells to activate T-cells, which cause malaria related inflammation [16].

The hypothesis of this study is that a deficiency of CXCL-10 in murine CM will enhance the production of Foxp3 (CD4+CD25+Foxp3+), IL-10 and IL-2, which will minimize or prevent fatal CM. To this end, experiments were conducted with CXCL-10-/- and wild type C57BL/6 mice and they were infected with *Plasmodium berghei *ANKA, a rodent malaria parasite commonly used as murine CM to ascertain the role of Foxp3, IL-10 and IL-2 in fatal CM pathogenesis.

## Methods

### Mice and *Plasmodium berghei *ANKA parasite

All studies were carried out with C57BL/6 wild type (WT) and CXCL-10 knock-out mice (CXCL-10-/-) aged 6-8 weeks. Mice were purchased from Jackson's laboratory (Bar Harbour, Maine USA), screened to ensure they were pathogen-free and were maintained at the Animal facility, at Morehouse School of Medicine on 12 h light/12 h dark cycle with access to water and food *ad libitum*. Stocks of *P. berghei *ANKA parasitized blood were obtained from Malaria Research and Reference Reagent Resources (MR 4) (Mansass, VA USA).

### Evaluation of parasitaemia and disease

Both WT and CXCL-10-/- mice were intraperitoneally injected with 10^6 ^*P. berghei *parasitized blood to cause murine CM. Control mice were sham injected with uninfected blood. Each batch consisted of equal numbers of infected and control mice. Parasitaemia was monitored by Giemsa-stained blood smears using light microscopy. Parasitaemia and survival of CXCL-10-/- and WT were evaluated. Mice were sacrificed at Days 2, 4 and 8, (post-infection p.i) to harvest brain, blood and spleen for analysis of murine CM induced markers. All procedures were performed in accordance with national regulations on animal experimentation and welfare, and the Care of Laboratory Animal Resources (CLAR) guideline was followed to minimize animal pain.

### Messenger RNA isolation and quantitative real-time RT-PCR analysis of Foxp3

#### RNA isolation and cDNA synthesis

Since CM involves both pathophysiology of the brain, the transcript levels of Foxp3 gene (specific marker for T-regs) was evaluated in mouse brain, and PBMC during *P. berghei *infection. Messenger RNA was isolated from infected and uninfected brain tissue and PBMC samples using Trizol reagent (Invitrogen, CA, USA) per manufacturer's instructions. Brain tissue samples were homogenized and PBMCs pelleted, and lysed by Trizol by repetitive pipetting. Chloroform was added to homogenates and incubated, followed by centrifugation at 12,000 g for 15 mins at 4°C. The aqueous phase obtained after centrifugation was pipetted into fresh tubes and RNA was precipitated by centrifugation at 12,000 g for 10 mins at 4°C in isopropanol. RNA samples were then washed, DNase treated and quantified. One microgram (1 μg) of DNase treated RNA was converted to cDNA using the Bio-Rad iScript cDNA synthesis kit (BioRad CA). Complementary DNA (cDNA) was synthesized by incubating the reaction mix at 25°C for 5 mins, 42°C for 30 mins and 85°C for 5 mins. cDNA samples were stored at -20°C until used.

#### Quantitative real-time RT-PCR analysis of Foxp3

Quantitative realtime-PCR was performed using the MyiQ real-time PCR detection system (Bio-Rad, CA USA). Polymerase Chain Reaction (PCR) was carried in a 50 μL reaction volume: 25 μL of iQ SYBR Green Supermix, 0.5 μL each of specific primer mix (100 nM as final concentration of each primer), 2 μL of cDNA and 19 μL of nuclease-free water.

Samples were run in duplicates in 96-well plate (Bio-Rad, CA USA) with Foxp3 specific primers, **( 5'-**GGCCCTTCTCCAGGACAGA-**3**'; **5**'GCTGATCATGGCTGGGTTGT-**3**') and -actin primers **(5**'-TGGGTCAGAAGGACTCCTATG-**3**', **5**'-CAGGCAGCTCATAGCTCTTCT-**3') **respectively.

### Protein isolation, SDS-PAGE and Western blot analysis of Foxp3

To determine if the expression of Foxp3 mRNA was translated into protein in the brain, protein was isolated from whole mice brains using lysis buffer (phosphate-buffered saline containing 0.05% Triton X-100, 0.15 M NaCl, 2 mM EDTA, 1 mM phenylmethylsulphonyl fluoride, and 20 μg aprotinin/ml). The total concentration of protein in the sample was determined by Lowry method, so that 30 μg of protein could be subjected to 12% SDS-PAGE. The separated protein was then blotted onto nitrocellulose membranes and probed with monoclonal antibody against mouse Foxp3 (Santa Cruz Biotechnology, Inc, CA USA) at a concentration of 1:2000, and anti-α-tubulin monoclonal antibodies (Sigma-Aldrich, MO, USA) as internal control at a concentration of 1:4,000. Goat anti-mouse IgG Horse Radish Peroxidase (HRP), BioRad, CA, USA) conjugate was used as a secondary antibody at 1:6000. A chemiluminescent substrate for the detection of HRP, (SuperSignal West Pico Chemilumiscent Substrate, PIERCE Thermo Fisher Scientific, IL, USA) was used to detect Foxp3 (48 kD) and α-tubulin (50 kD) proteins in mouse brain.

### Preparation of plasma samples from blood

In order to evaluate the induction of IL-10 and TGF- β1, and Th1 cytokine IL-2 during *P. berghei *infection, heparinized blood samples from infected and uninfected mice were collected. Blood was centrifuged for 15 mins at 3,000 g and plasma samples were collected and stored until used.

### Isolation of peripheral blood mononuclear cells (PBMC) from spleen

The spleen is an important lymphoid organ where T-cell induction occurs during malaria infection, and is also involved in the destruction of parasitized RBCs in infected mice. Therefore, to obtain induced T-cells during *P. berghei *infection, PBMC were obtained from spleen, and T-cells purified for further analysis. Briefly, spleens from infected and uninfected mice were collected at Days 2, 4 and 8 post-infections. Using a 70 μm cell strainer (BD Bioscience CA USA) and plunger of syringe, splenic cells were teased through the cell strainer into a petri dish, keeping the cell strainer suspended in complete RPMI 1640 (10% FBS, 1% L-glutamine and Penicillin-streptomycin). Cells were collected and centrifuged at 1200 rpm for 5 minutes at 4°C and pellets were collected. Red blood cells in pellets were lysed with RBC lysis buffer (Sigma, MO USA). Peripheral blood mononuclear cells (PBMCs) pellets were resuspended in complete RPMI 1640 medium until used.

### Isolation of CD4+CD25+ regulatory T cells from splenic PBMC

Regulatory T-cells (CD4+CD25+) were isolated from splenic PBMC using the Miltenyi magnetic beads and columns system according to manufacturer's instructions (Miltenyi Biotec Inc. CA USA). Regulatory T cells were isolated in a two-step procedure. First, non-CD4^+ ^T-cells, CD8^+^, γ/δ, B cells, NK cells, dendritic cells, monocytes, granulocytes, and erythroid cells, were indirectly labeled magnetically by using a cocktail of biotin-conjugated antibodies at the appropriate titers according to manufacturer's recommendation and were magnetically labelled with anti-Biotin microbeads for depletion. Subsequently, cells were processed with labelled CD25 microbeads to purify CD4+CD25+ T-cell population from cell suspension. CD4+ CD25+ and CD4+CD25- T- cells were purified with mouse anti-CD25-PE antibody and anti-PE microbeads according to manufacturer's recommendation. Cell numbers and viability were determined. Purified CD4+CD25+ (>95% purity, data not shown) were suspended in complete RPMI 1640 medium until used.

### Preparation of *Plasmodium berghei *ANKA antigens for restimulation assay 	

*Plasmodium berghei *ANKA crude antigens were prepared per the method of Kima and colleagues [17]. *Plasmodium berghei *ANKA parasitized RBCs were washed twice with Hanks Balanced Salt Solution (HBSS). Cells were centrifuged at 1,100 × g for 10 mins to remove leukocytes (buffy coat). Cells were diluted to a concentration of 10^9 ^PRBC/mL and were subjected to three freeze and thaw cycles. Cells were centrifuged at 100,000 × g for 1 h. Cells from uninfected red blood cell controls were subjected to the same procedure. Supernatants containing crude antigen were quantified using the Quant-IT™ protein assay kit with the Qubit fluorometer (Invitrogen, USA), and frozen at -20°C until used. Crude protein lysate at 50 μg/mL was used to restimulate purified cells *in vitro*.

### *In vitro *restimulation of CD4+CD25+ and CD4+CD25-T cells with *P. berghei *ANAKA parasite antigens

High mRNA levels of Foxp3 in PBMC collected at day 4 using the qRT-PCR analysis showed the highest levels of CD4+CD25+Foxp3+, hence CD4+CD25+ and CD4+CD25-T-cells were purified from infected and control spleens at Day 4 from both WT and CXCL-10-/- mice. They were restimulated *in vitro *with *P. berghei *antigens to determine and compare cellular production of CD4+CD25+Foxp3+ associated anti-inflammatory cytokines IL-10 and TGF-β1. To this end, 5 × 10^5 ^cells/well were cultured with *P. berghei *antigens (50 μg/mL) and dendritic cells as antigen presenting cells (APC). Cells cultured with supernatants prepared from uninfected red blood cells were used as negative controls. Cells were cultured for 5 days. Six hours (6 hrs) before harvesting, phorbol-12-myristate-13-acetate (PMA) (50 ng/mL) and ionomycin (1 μg/mL) were added to cultures to induce the production of cytokines to measurable levels *in vitro*. Cultured cells were harvested and centrifuged and the supernatants frozen at -20°C until they were used.

### Analysis of IL-10, IL-2 and TGF-β1 in plasma and cell culture supernatants

The levels of IL-10, TGF-β1 and IL-2 in plasma samples collected from heparinized blood and supernatants from *P. berghei *antigen restimulated CD4+CD25+ and CD4+CD25-T-cells were determined using a quantikine ELISA kit (R&D systems (MN USA) per manufacturer's instructions. Interleukin-10, TGF-β1 and IL-2 detection methods were similar except that for TGF-β1 detection, plasma and supernatants were activated with 10 μL of 1NHCL/40 μL of plasma or supernatant for 10 minutes, and subsequently neutralized with 8 μL of 1.2 N NaOH/0.5HEPES. Sample mixture was diluted 60-fold according to manufacturers' instructions prior to commencing the assay.

Briefly, 50 μL samples, controls and standards were prepared and added to the respective IL-10, TGF-β1 and IL-2 antibody coated microplate wells. After incubation and washing, 100 μL of IL-10, TGF-β1 and IL-2 conjugates were added to each well. Samples were washed again and substrates were added to the complex and incubated further. The reaction was stopped and optical densities were read at 540 nm. Duplicate readings of samples were averaged and the averages of zero standard optical densities were subtracted. A standard curve was plotted and the concentrations of IL-10 and TGF-β1 in plasma and supernatants and IL-2 in plasma were evaluated. Because samples for TGF-β1 detection have been diluted 60-fold in the activation step prior to the assay, the measured concentrations were multiplied by the dilution factor (60) to obtain the actual concentration of TGF-β1 in the sample.

### Statistical analysis

Statistical differences between Foxp3, IL-10 and IL-2 production in *P. berghei-*infected and uninfected as well as between infected CXCL-10-/- and WT mice were determined by student two-tailed test, and p < 0.05 was considered statistically significant. Graph pad prism statistical software version 5.0 was used for all analysis.

## Results

### *Plasmodium berghei *ANKA infection in CXCL-10-/- and WT mice

**Figure 1:**
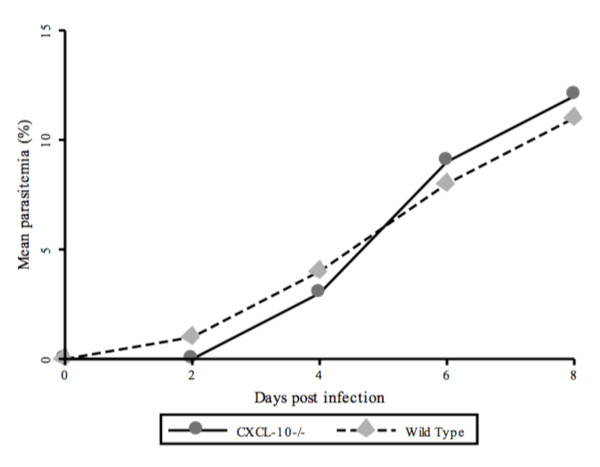
**Parasitaemia in *Plasmodium berghei *ANKA (10^6^) infected Wild type and CXCL-10-/- C57/BL6 mice at days 2, 4, 6 and 8 post-infection**. Parasitaemia in infected wild type and CXCL-10-/- was monitored daily by Giemsa-stained blood smears collected from the tail vein using light microscopy and counting at least 300 thin film of red blood cells. Mice were sacrificed at Days 2, 4 and 8, (post-infection p.i) to harvest brain, blood and spleen for analysis of murine CM induced markers.

During infection with *P. berghei*, WT mice exhibited characteristic symptoms as expected of murine CM including ruffled furs, weakness and ataxia compared with the CXCL-10-/- mice. However, there was no significant difference in parasitaemia between CXCL-10-/- and WT mice (Figure 1).

### Foxp3 mRNA expression is up-regulated in brain in *P. berghei*-infected CXCL-10-/- than in WT mice

**Figure 2:**
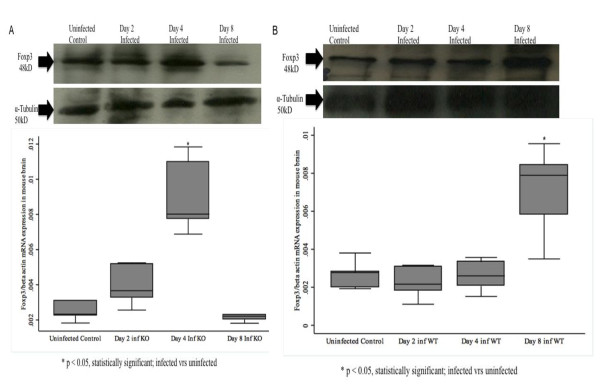
**qRT-PCR and Western Blot analyses of Foxp3 in CXCL-10-/- and WT mouse brain**. CXCL-10-/- and wild type C57BL/6 (n = 5-10) were infected with *P. berghei *ANKA parasitized blood intra-peritoneally. Control mice were injected with uninfected blood. Infected and uninfected mice were sacrificed at day 2, 4 & 8. Messenger RNA and protein were isolated from brain tissue samples, and qRT-PCR and Western blot were performed using the MyiQ real-time PCR detection system (Bio-Rad, CA USA) with primers for Foxp3 and -actin as internal control and SDS-PAGE Western blot and Foxp3, and α-tubulin antibodies respectively. *P. berghei *ANKA induced Foxp3 mRNA and protein expressions at day 4 in CXCL-10-/- brain were higher and statistically significant compared with the expression in WT. At day 8 p.i however, *P. berghei *ANKA induced Foxp3 mRNA and protein were higher in WT brain than in CXCL-10-/. *P. berghei *ANKA induced early expression of Foxp3 mRNA and protein in CXCL-10-/- than in WT.

Forkhead transcription factor 3 (Foxp3) mRNA was significantly elevated at Day 4 post-infection (p < 0.05) when compared with control mice (Figure 2A). WT mice showed no difference in Foxp3 mRNA expression between *P. berghei*-infected and control mice in brain at day 2 and 4 post-infection (Figure 2B). Foxp3 mRNA expression was higher in *P. berghei-*infected in CXCL-10-/- mice at Day 2 and 4, and later at day 4 (p < 0.05) in WT mice (Figure 2C).

### Foxp3 protein expression in mouse brain correlates with mRNA expression during *P. berghei *ANKA infection in CXCL-10-/- and WT

Western Blots analysis of Foxp3 protein expression indicated that Foxp3 proteins (48 kD) in infected CXCL-10-/- peaked at day 4 at higher level than in uninfected controls at day 2 and 8 post-infection (Figures 2A and 2B). In WT mouse brain, Foxp3 protein expression was significantly elevated at day 8 post-infection than at days 2 and 4, and in uninfected controls (Figure 1B). There was a direct relationship between mRNA and protein expression of Foxp3 in mouse brains of both CXCL-10-/- and WT during *P. berghei *ANKA infection (Figures 1A and 1B). The expression of α-tubulin (50 kD) ('housekeeping gene', for internal control) was similar in all sample analysed (Figures 2A and 2B).

### Foxp3 mRNA expression is up-regulated in PBMC of *P. berghei*-infected CXCL-10-/- than in WT mice

**Figure 3:**
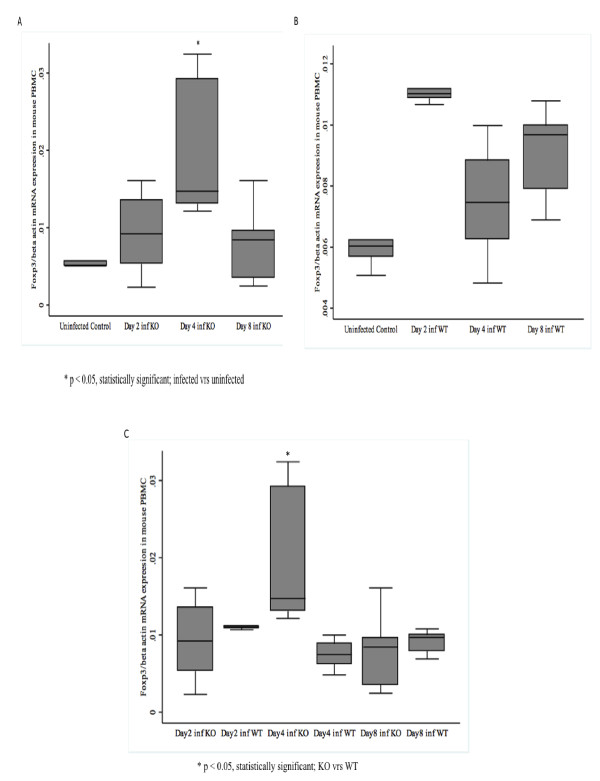
**(A-C): qRT-PCR analysis of Foxp3 in CXCL-10-/- and WT mouse peripheral blood mononuclear cells (PBMC)**. CXCL-10-/- and wild type C57BL/6 (n = 10) were infected with *P. berghei *ANKA parasitized blood intra-peritoneally. Control mice were injected with uninfected blood. Mice were sacrificed at day 2, 4 & 8. Messenger RNA was isolated from brain tissue samples, DNase treated and quantified. One microgram (1 μg) of DNase treated RNA was converted to cDNA and qRT-PCR was performed using the MyiQ real-time PCR detection system (Bio-Rad, CA USA) using primers for FOXP3 and -actin as internal control. *P. berghei *ANKA induced Foxp3 mRNA expression at day 4 in CXCL-10-/- PBMC was higher and statistically significant compared with WT (Figure 3C).

Foxp3 mRNA increased in PBMC of infected CXCL-10-/- mice at day 2 and 4 greater than control, declining by day 8 p.i. Interestingly, increased in Foxp3 mRNA in PBMCs of infected WT at day 2, 4 and 8 were not statistically significant when compared with it corresponding PBMC controls (Figure 3B). Foxp3 mRNA in PBMC of infected CXCL-10-/- at day 4 was significantly up-regulated (~88%) compared with infected WT mice (Figure 3C). There was no differences in Foxp3 mRNA expression between infected PBMCs of CXCL-10-/- and WT at day 2 and 8 (Figure 3C). In all mice, *P. berghei *infection induced significant amount of Foxp3 mRNA expression in brain and PBMCs in CXCL-10-/- at day 4 post-infection when compared with WT.

### *Plasmodium berghei *ANKA induces early production of IL-10 in CXCL-10-/-

**Figure 4:**
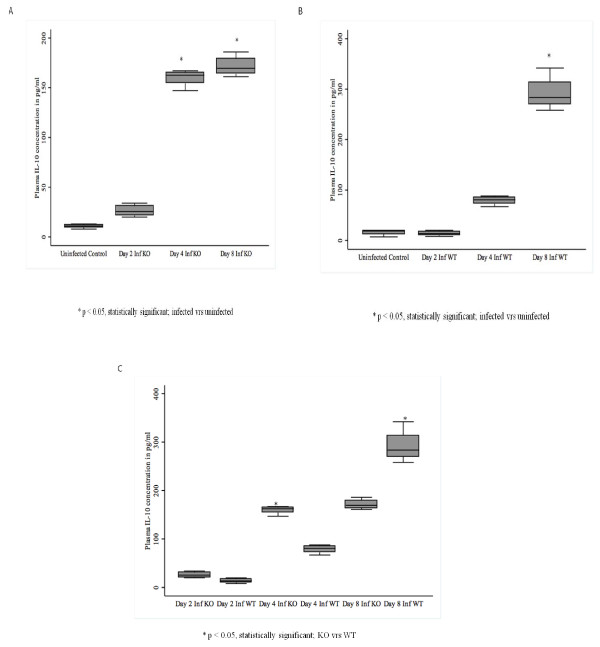
**(A-C): Plasma levels of IL-10 in *P. berghei *ANKA infected and control CXCL-10-/- and wild type C57BL/6 mice**. CXCL-10-/- and wild type C57BL/6 (n = 10) were infected with *P. berghei *ANKA parasitized blood intra-peritoneally. Control mice were injected with uninfected blood. Infected and uninfected mice were sacrificed at day 2, 4 & 8. Plasma was collected and ELISA was performed to determine IL-10 levels using the R&D systems (MN USA). IL-10 in plasma of infected mice was higher than in uninfected mice except on day 2 in infected WT (Figures 4A & 4B). Plasma level of IL-10 in infected CXCL-10-/- is significantly up-regulated compared with infected WT at day 2 and 4 post-infection (Figure 4C). Interestingly, IL-10 at day 8 pi in WT plasma was significantly higher in WT than in CXCL-10-/-.

Plasma production of IL-10 in plasma at days 4 and 8 in both infected CXCL-10-/- and WT mice was elevated when compared with controls (Figures 4A and 4B), indicating that parasites may be responsible for altering IL-10 levels in both WT and CXCL-10-/- mice. Interleukin-10 levels were higher at Day 4 in infected CXCL-10-/- than was observed for WT. In CXCL-10-/-, IL-10 levels did not change significantly by Days 4 and 8. Infected WT mice, on the other hand, had higher levels IL-10 at day 8 than at day 4 (Figure 4B). These observations suggest that IL-10 levels increased about 250% from day 4 to day 8 (late stage) post-infection in WT compared to what occurred from day4 to day8 in CXCL-10-/-.

### IL-2 in CXCL-10-/- mice is elevated in *P. berghei *infected mice

**Figure 5:**
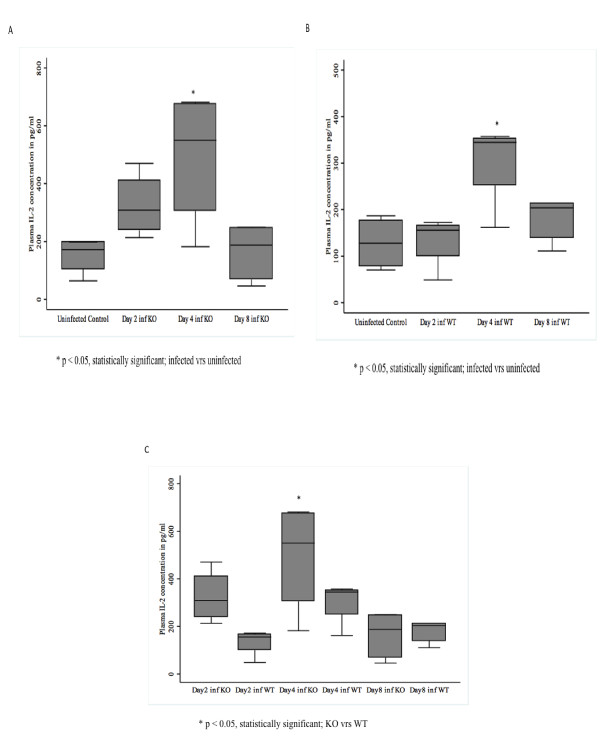
**(A-C): Plasma levels of IL-2 in *P. berghei *ANKA infected and control CXCL-10-/- and wild type C57BL/6 mice**. CXCL-10-/- and wild type C57BL/6 (n = 10) were infected with *P. berghei *ANKA parasitized blood intra-peritoneally. Control mice were injected with uninfected blood. Mice were sacrificed at day 2, 4 & 8. Plasma was collected from infected and uninfected mice and ELISA was performed to determine IL-2 levels using the R&D systems (MN USA). There was significant induction of IL-2 in CXCL-10-/- than in WT at day 2 (statistically significant, p < 0.05) and 4 post-infection. Differences between IL-2 levels in CXCL-10-/- and WT at day 8 were not significant (error bar on plot for day 8 CXCL-10-/- (KO) could be a potential outlier. It presence or removal did not affect the difference between the two group of mice during the analysis).

Induction of IL-2 in peripheral blood started early at day 2 and was significantly elevated (p < 0.05) at day 4 post-infection compared with uninfected controls in CXCL-10-/- mice (Figure 5A). Levels of IL-2 declined at day 8 post-infection.

Levels of IL-2 during *P. berghei *infection in WT mice began at day 4 and was higher when compared with IL-2 expression at days 2, 8 as well as uninfected controls (Figure 5B). It was observed that there is a significant induction of IL-2 in CXCL-10-/- than in WT, at day 2 (~40%,) and 4 (~80%, p < 0.05) post-infection. There was no significant differences between IL-2 levels in CXCL-10-/- and WT at day 8 (Figure 5C).

### *Plasmodium berghei *ANKA antigen induces CD4+CD25+ and CD4+CD25- T cells to produce IL-10

**Figure 6:**
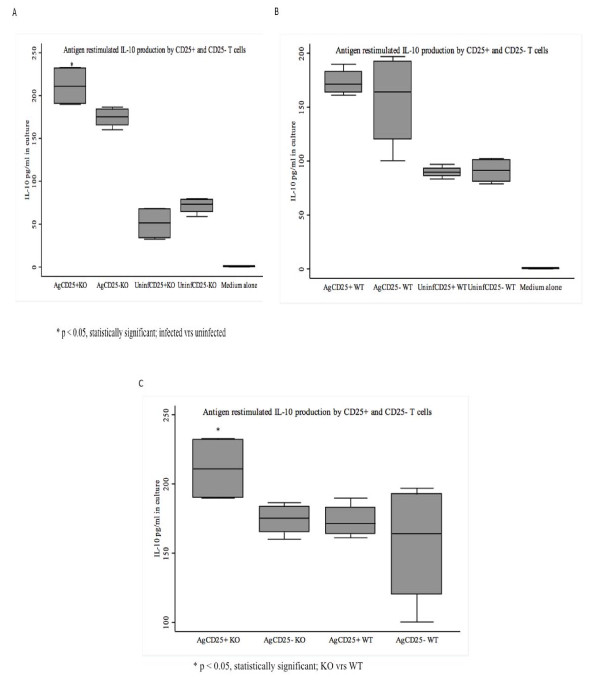
**(A-C): IL-10 production in *P. berghei *antigen re-stimulation of CD4+CD25+ and CD4+CD25- T cells from CXCL-10-/- and wild type C57BL/6 mice**. Day 4 infected mice (n = 6) spleens were harvested and peripheral blood mononuclear cells were isolated. Regulatory T cells (CD4+CD25+) and regulatory T cells-depleted T cells (CD4+CD25-) were isolated using the Miltenyi Biotec Inc (CA, USA) isolation kit. Cells (5 × 10^5^cells/well) were re-stimulated with *P. berghei *antigens (Ag) (50 μg/ml) plus PMA and ionomycin. Controls consisted of cells re-stimulated with supernatant from uninfected red blood cells (Uninf.). Cells were cultured for 5 days and IL-10 in harvested supernatants was determined by ELISA. There was significant difference in IL-10 production between antigen (Ag) restimulated cells than in controls (Uninf) (Figures 6A and 6B). Interestingly there was no significant difference in IL-10 levels between antigen (Ag) restimulated cells from CXCL-10-/- and WT (Figure 6C).

Cells from WT and CXCL-10-/- mice stimulated with *P. berghei *antigens produced higher amounts of IL-10 than cells stimulated with supernatants from uninfected cells in RPMI 1640 medium alone (Figures 6A and 6B). Figure 6C indicates that CXCL-10-/- CD4+CD25+ T-cells IL-10 production was higher (~30%) and statistically significant (p < 0.05) than that of WT. There were no differences in IL-10 production between CD4+CD25- from CXCL-10-/- and WT mice (Figure 6C). The ELISA assay could not detect differences in TGF-b1 protein production between CXCL-10-/- and WT.

## Discussion

Although several mechanisms have been proposed to explain cerebral malaria immunopathogenesis, the precise mechanisms is still unknown. Dysregulation of homeostasis of pro- and anti- inflammatory factors has been linked to the severity of CM. For instance, over production of pro-inflammatory cytokines resulted in dysfunction of the blood-brain barrier (BBB) leading to CM complications [18-21].

Recently, a number of chemokines and their corresponding receptors were implicated in CM pathogenesis [2-4]. Studies conducted in our laboratory and elsewhere using human CM samples from Ghana and India revealed that excessive production of CXCL-10 in severe malaria patients is an important predictor of mortality risk in CM [4,21]. These studies demonstrated that there were significant increase in pro-inflammatory markers including interleukin-8 (IL-8), interleukin-1 receptor agonist (IL-ra), interferon-inducible protein-10 (CXCL-10), macrophage inflammatory protein-1beta (MIP-1β), soluble tumor necrosis factor receptor-1 and 2 (sTNF-R1 and sTNF-R2) in patients who died of CM [5].

Interferon gamma inducible protein-10 (CXCL-10) levels in serum were independently associated with fatal CM [4,21]. Thymus (T) cells produce CXCL-10, and, therefore, a significant up-regulation of CXCL-10 in sera of patients dying of CM indicates a high level of activation of T-cells associated with CM. CXCL-10 is a member of the CXC pro-inflammatory chemokines and binds the CXCR3 receptor which is expressed by activated T-cells [22]. Thymus (T) cells play a major role in CM immunopathogenesis although the mechanism by which their regulation modulates CM severity is not understood [23,24]. Mice without T-cells do not develop CM and CM is attenuated by blocking CD4+ and CD8+ T- cells [1]. Thus it is logical that blocking T-cells or interfering with T-cell activation will decrease risk of fatal CM.

In this study CD4+CD25+Foxp3+ transcription factor and specific cell marker Foxp3 mRNA and protein in *P. berghei*-infected mice was up-regulated in mouse brain. Brain and PBMC Foxp3 expression induced by *P. berghei *were significantly up-regulated in CXCL-10-/- mice at Day 4 post-infection compared with WT. This suggests an elevated systemic expression of Foxp3 mRNA and protein in CXCL-10-/- than in WT mice during the infection. This observation is in line with recent human studies conducted in The Gambia in West Africa where individuals living in areas with high malaria transmission had higher levels of regulatory T-cells compared with those living in areas where malaria is rare [25]. However this contrasts with other studies, which reported a minor increase in Foxp3 expression during *P. berghei *infection at Day 7 post-infection [26] (early expression of Foxp3 was not reported in this article).

Regulatory T cells are key cerebroprotective immunomodulators during acute strokes in murine model. Absence of CD4+CD25+Foxp3+ enhanced activation of resident and invading inflammatory cells (microglia and T-cells) to produce higher levels of TNF and IFN-γ, which are deleterious to the brain [27]. In this study, it was determined that early up-regulation of Foxp3 (mRNA and protein) in the brain and PBMCs correlated positively with CM protection in CXCL-10-/- mice than in WT. Regulatory T-cells induced CD4+ T-cell apoptosis during the early stage of *P. yoelii *17XL infection and this in effect regulates pro-inflammatory responses [28]. Therefore, up-regulation of Foxp3 in the brain may inhibit over activation of microglia and T-cells which could prevent parasite associated complications in the brain. CXCL-10 itself has been associated with fatal CM and it absence has been linked to enhanced CD4+CD25+Foxp3+ expression and activation leading to its ability to dampen over activation and production of pathogenic inflammatory factors which cause CM. These taken together could be responsible for the inhibition of CM outcome in the CXCL-10-/- compared with the WT in our study, compared with the study in reference 29.

Some reports indicate that expansion of CD4+CD25+Foxp3+ during murine malaria did not prevent murine CM [29]. However, other studies have shown that CD4+CD25+Foxp3+ suppressed CD4+ T-cell function and inhibited the development of murine CM in *Plasmodium berghei*-specific Th1 response [30, 31]. Increase in numbers and activation of CD4+CD25+Foxp3+ control the production of pro-inflammatory cytokines and mediate a counter-regulatory response to overwhelming inflammation during lethal *Plasmodium chabaudi adami *infection and did not contribute to parasite immune evasion [32]. Significant up-regulation of Foxp3 mRNA and protein during *P. berghei *infection in CXCL-10-/- in this study is associated with protection against murine CM; a first report of the role of Foxp3 gene and protein expression in severity of CM in CXCL-10-/- mice.

It was observed in this study that IL-10 levels increased in plasma during *P. berghei *infection in CXCL-10-/- than in wild type mice. Interleukin-10 (IL-10) is an anti-inflammatory cytokine and thus increased production during parasite infection was necessary to ameliorate the disease outcome. Interleukin-10 (IL-10) levels in infected CXCL-10-/- at Days 2 and 4 were higher than in infected WT mice. Possibly, early induction of IL-10 and Foxp3 combined to provide anti-inflammatory responses to mediate the cerebroprotective effects against CM in CXCL-10-/- mice as evidenced in the absence of CM symptoms in those mice. Regulation of proinflammatory response during murine malaria infection is mediated by regulatory T- cells in an IL-10 dependent manner [28]. Additionally, CD4+CD25+Foxp3+ have recently been reported as major cerebroprotective modulators of post-ischemic inflammatory brain damage with IL-10 as the predominant associated anti-inflammatory cytokine modulating this effect [27]. Therefore, IL-10 producing CD4+CD25+Foxp3+ activated early in a robust inflammatory setting through a feedback mechanism would consequently reduce severity of CM.

In this study interleukin-2 cytokine was up-regulated in CXCL-10-/- mice than WT. Interleukin-2 (IL-2) is a Th1 cytokine produced by activated T lymphocytes and plays a key role in promoting the expansion of antigen-specific T cells and also mediates multiple immune responses on a variety of cell types including thymocytes, activated B cells, monocytes, natural killer cells and oligodendrocytes. IL-2 mediates signaling through IL-2Rα, and regulates Foxp3 expression in CD4+CD25+Foxp3+ [33,34]. Induction of Foxp3 during *Plasmodium falciparum *infection is driven by IL-2 [34]. The results showed that at days 2 and 4, IL-2 was significantly up-regulated in plasma of CXCL-10-/- than in WT, indicating that the absence of CXCL-10 promotes IL-2 expression during murine malaria and that it could play an important role in the pathogenesis of severe malaria.

Thymus (CD4+, and CD25+ T cells) were the major cellular source of IL-10 and TGF-b1 cytokines in malaria infected red blood cells (iRBC) when co-cultured with PBMCs [34]. CD4+CD25+ and CD4+CD25- T-cells cultured with *P. berghei *antigens secreted more of IL-10 than cells cultured with non- *P. berghei *supernatant and RPMI 1640 medium alone confirming that *P. berghei *parasite antigens were inducing IL-10 production. It was observed that IL-10 production by CD4+CD25+ T-cells from CXCL-10-/- was significantly higher than wild type mice *in vitro *(Figures 6A, 6B and 6C), implying that CD4+CD25+ T-cells is a potential source of IL-10 during malaria infection.

Taking all of the above observation together, it could be concluded that, high levels of IL-2 in CXCL-10-/- mice during *P. berghei *ANKA infection induce CD4+CD25+Foxp3+ measured by levels of Foxp3 transcription and translation. Subsequently it seems that activated CD4+CD25+Foxp3+ produce high levels of IL-10, and are able to mount a robust anti-inflammatory response, which counter the inflammatory effects of *P. berghei *parasite thus preventing fatal CM in CXCL-10-/-.

## Conclusion

*Plasmodium berghei *ANKA infection in CXCL-10-/- mice induces early production of T-regs expressing Foxp3 in brain and PBMC, and IL-10 and IL-2 than in WT. Also, CD4+CD25+ T cells from CXCL-10-/- mice produce high levels of IL-10 than WT. CXCL-10 involvement in the pro-inflammatory networks coupled with early activation of Foxp3 (CD4+CD25+Foxp3+) in conjunction with IL-10 and IL-2 are important mediators of fatal murine CM, and, therefore, strategies to regulate their expression will have the potential to prevent fatal CM.

## Abbreviations

BBB: (Blood-Brain Barrier); cDNA: (complementary DNA); CM: (cerebral malaria); CTLA: (cytotoxic T lymphocyte antigen); CXCL-10: (interferon-gamma inducible protein 10); ECM: (experimental cerebral malaria); ELISA: enzyme-linked immunosorbent assay; HBSS: (Hanks Balanced Salt Solution); IFN: (interferon gamma); IL: (interleukin); iRBC: (infected red blood cells); KO: (knock out); mRNA: (messenger RNA); RPMI: (Roswell Park Memorial Institute); RT-PCR: (reverse transcription PCR); TGF: (tumor growth factor); TNF: (tumor necrosis factor); WT: (wild type).

## Competing interests

The authors declare that they have no competing interests.

## Authors' contributions

SBY conceived the idea, designed the study, performed sample collection and all experimental procedures, data analysis and writing of the manuscript. WNO participated in the performance of immunoassay and data analysis, BVC, participated in study design, coordination and review of manuscript, SJK participated in study design and coordination, supervised the study, and revised the manuscript for important intellectual content. All authors read and approved the final manuscript.
